# Literary Note

**Published:** 1909-10

**Authors:** 


### LITERARY NOTE

The September number of the *Trained Nurse and Hospital Review,* the pioneer nursing magazine of America, now over 21 years old, contains a number of articles not only of interest to nurses, physicians, and hospital authorities, but also valuable and instructive to all who are concerned in promoting the national, social, economic, or ethical welfare.

